# Perceptions and Utilization of Registered Dietitian Nutritionists in Multiple Sclerosis Care: A Pilot Survey of Multidisciplinary Providers †

**DOI:** 10.3390/nu17030385

**Published:** 2025-01-22

**Authors:** Olivia Wills, Alaina Bradford, Mona Bostick, Yasmine Probst, Tyler J. Titcomb

**Affiliations:** 1 School of Medical, Indigenous and Health Sciences, University of Wollongong, Wollongong, NSW 2500, Australia; ow873@uowmail.edu.au (O.W.); yasmine@uow.edu.au (Y.P.); 2 Department of Neurology, UC Health, Cincinnati, OH 45219, USA; 3 Independent Researcher, Greensboro, NC 27401, USA; 4 Departments of Epidemiology and Internal Medicine, University of Iowa, Iowa City, IA 52242, USA

**Keywords:** multiple sclerosis, registered dietitian nutritionists, multidisciplinary care, survey

## Abstract

Background/Objectives: Registered dietitian nutritionists (RDNs) are allied healthcare professionals who can help people with multiple sclerosis (MS) incorporate healthy eating, but little is known about their involvement in MS care. Thus, the objective of this survey was to investigate the perceptions and utilization of RDNs in MS care among multidisciplinary MS providers in the United States and Canada. Methods: An online survey was disseminated via the Consortium of MS Centers email listserv and MS-specific scientific conferences. The survey queried practitioner type, RDN referrals, the perceived benefits of RDNs in MS care, and the proportion of their patients who follow ‘MS diets’ or have nutrition-related issues. Reasons for or against RDN referral and beneficial resources were also queried. Results: Of the 60 completed surveys, respondents were primarily neurologists (*n* = 27, 45.0%). Most (*n* = 43, 71.7%) indicated that half or more of their patients inquire about diet, but *n* = 32 (53.3%) indicated that very few follow an ‘MS diet’ and *n* = 47 (78.3%) indicated that very few decline disease-modifying therapies to follow an ‘MS diet’. Most (*n* = 45, 77.6%) respondents indicated referring their patients to a RDN with lack of nutrition knowledge/general healthy eating advice (*n* = 34, 73.9%) and overweight/obesity (*n* = 31, 67.4%) as being the most common reasons for referral. RDNs were reported as being helpful or extremely helpful by *n* = 38 (84.4%) of respondents who reported referring to RDNs. Most (*n* = 46, 79.3%) indicated that their patients would benefit from having an RDN with MS-specialized training as a member of staff. Conclusions: MS care providers support the need for RDNs with specialized training in MS care.

## 1. Introduction

People with multiple sclerosis (MS) show a great interest in utilizing dietary modifications to improve their health, self-manage symptoms, and improve quality of life [1]. Surveys consistently observe that up to half of people with MS report making dietary modifications [2,3,4]; however, dietary recommendations are not part of usual MS care. International evidence suggests that people who are newly diagnosed with MS alarmingly receive little advice or support for changing their diet from their neurology care team [5] and the advice they do receive is often inconsistent [6]. Furthermore, neurologists report not having adequate time to discuss diet with their patients with MS [7]. As such, it is common for people with MS to seek information on diet(s) from online sources [8] that may lack credibility and evidence-based recommendations [9]. In fact, diet is the most frequently searched self-management strategy on the internet by people with MS [10].

MS-specific dietary recommendations and behavioral change support are highly desired by people with MS [10,11,12,13]; however, the current state of evidence does not support any specific diets for MS care [14,15]. Network and pairwise meta-analyses found that several dietary interventions reduce fatigue and improve physical and mental quality of life, but the credibility of evidence is considered very low due to methodological issues of the included trials [16,17]. Interestingly, both studies identified trials that explored the effects of specific diets (e.g., ketogenic and Paleolithic), and neither identified studies that investigated conventional healthy eating recommendations. The network meta-analysis observed that only specific diets recommending an increased intake of fruit and vegetables, and a decreased intake of ultra-processed foods (typical conventional healthy eating recommendations), were associated with favorable outcomes [17]. Furthermore, a recent systematic review of observational studies observed that dietary patterns rich in vegetables, fruit, legumes, whole-grains, fish, and fiber (consistent with conventional healthy eating recommendations) were associated with a higher quality of life and reduced disability among people with MS [18].Together, these results led members of the National MS Society Nutrition Workgroup to recommend the inclusion of registered dietitian nutritionists (RDNs) in usual MS care to help people with MS implement evidence-based, healthy diet recommendations to improve wellbeing and prevent cardiometabolic comorbidities [19]. These recommendations were also recently replicated in a Delphi consensus of lifestyle behaviors to promote brain health-focused care for multiple sclerosis [20].

At the time of diagnosis, people with MS have a high prevalence of several cardiometabolic comorbidities, including type 2 diabetes mellitus and hypertension; however, paradoxically, they are less likely to receive treatment for these comorbidities compared to people without MS [21]. RDNs are accredited allied health professionals who have expertise in nutrition and provide evidence-based medical nutrition therapy for many of the cardiometabolic comorbidities that are associated with worse MS outcomes [22]. Evidence regarding the effect of nutrition therapy provided by RDNs on MS outcomes remains sparse; however, preliminary evidence shows that it increases diet quality, improves micronutrient intake, and improves anthropometric measures [23,24,25]. Furthermore, little is known about the frequency of involvement of RDNs in MS care, nor the perceptions of MS care providers regarding the benefit of RDNs in MS care. An analysis of the North American Research Committee on MS patient registry in 2003 found that 24.2% of 20,778 participants reported seeing an ‘RDN or nutritionist’ in the previous 12 months [26]; however, this survey did not distinguish between RDNs and other non-accredited ‘nutritionists’. Cross-sectional surveys of people with MS observed that 16.3% reported using an RDN as their primary source of nutrition information [27], and 11.6% reported making dietary changes based on advice from an RDN [28]. However, over a 10-year follow-up period, only 8% of people with MS reported seeing a RDN in the Stockholm MS study [29]. Furthermore, a 2022 survey of multidisciplinary MS care providers in Canada observed that respondents reported referring 10% of their patients with MS to RDNs, and over 60% indicated that RDNs are important or very important to MS care [30]. However, the reasons for and for not referring patients with MS to RDNs among MS care providers remain unknown.

Therefore, the objective of this pilot survey was to investigate the perceptions and utilization of RDNs in MS care among a sample of multidisciplinary MS care providers.

## 2. Materials and Methods

This 6-item pilot survey was designed by members of the RDN Special Interest Group of the Consortium of MS Centers (CMSC) to understand the perceptions and utilization of RDNs among multidisciplinary MS care providers based on the research team’s expertise and experiences. Because time and survey burden are well-established barriers to survey completion reported by physicians [31], this survey was intentionally designed for brevity to increase completion rates [32]. The survey was determined as ‘not human subjects research’ by the University of Iowa Institutional Review Board (#202304271), as no identifiable information was collected. Prior to dissemination, the survey was assessed for face validity by members of the CMSC RDN Special Interest Group (*n* = 7) and CMSC administration (*n* = 4) for item wording, recall period, and response options to ensure the acceptability and appropriateness of the survey for the target sample and outcomes. Where appropriate, questions and response options were revised prior to dissemination to MS care providers.

The survey included multiple choice questions to obtain respondents’ professional discipline, if they have ever referred a patient with MS to an RDN, and how beneficial they perceive RDNs to be in the care of their patients with MS. Additionally, the survey included multiple choice questions inquiring about the proportion of their patients with MS who inquire about diet, follow ‘MS diets’ (e.g., Best Bet, OMS, Swank, Wahls, etc.), refuse disease modifying therapies due to the perception that MS can be managed by diet, have any cardiometabolic comorbidities, and have nutrition-related issues (e.g., dysphagia, constipation, malnutrition, etc.). In addition, the survey included checkbox questions to identify reasons for referring their patients with MS to RDNs, and what resources the providers or their patients would benefit from at their clinical practice were queried. Finally, respondents who reported not referring to RDNs were asked to report their reasons for not referring. The complete survey can be found in Supplemental S1.

The survey was sent to the CMSC email listserv twice, once as a standalone email (July 2023) and once as part of a monthly newsletter (August 2023). The CMSC listserv consists entirely of neurologists, nurses, and other multidisciplinary allied healthcare professionals (*n* = 275) and institutional (*n* = 1490) members located in the United States and Canada, ensuring that surveys were sent only to MS care providers and administrative staff at MS centers. To obtain a larger sample size, a QR code was displayed during poster sessions at the Americas Committee for Treatment and Research in Multiple Sclerosis (ACTRIMS) Forum and CMSC annual meetings in March and June 2024, respectively [33].

Descriptive statistics were calculated as frequency (%). Differences in desired beneficial resource responses and responses between neurologists and other MS care providers were assessed with chi-square tests of independence using SAS software (version 9.4; SAS Institute, Inc.; Cary, NC, USA). A *p*-value of ≤ 0.05 was considered statistically significant.

## 3. Results

The survey was emailed to 1765 unique email addresses of the CMSC listserv and had a low response rate of 2.1% (37/1765). An additional 37 responses were obtained from the 2024 CMSC and ACTRIMS Forum annual meetings for a total of 74 responses. After excluding respondents who consented but did not complete the survey (*n* = 13) and who reported not being a healthcare provider (*n* = 1), a total of 60 respondents were included in the analysis with a completion rate of 81.1% (60/74). Respondents were primarily neurologists (*n* = 27; 45.0%) followed by nurses (*n* = 8; 13.3%), and nurse practitioners (*n* = 6; 10.0%; Table 1).

**Figure 1:**
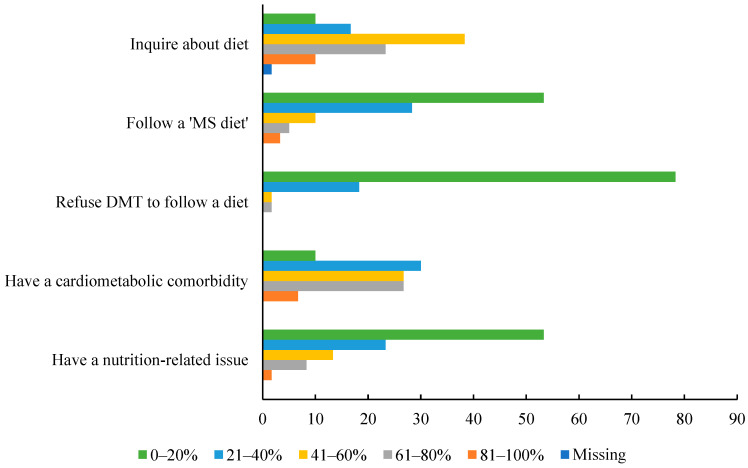
Reported approximate proportions of patients with MS exhibiting diet-related characteristics. Bars of different colors represent the percent of respondents selecting the corresponding option. Abbreviations: disease-modifying therapy, DMT; multiple sclerosis, MS.

When asked what proportion of their patients with MS inquire about diet, 41–60% was the most frequently selected option by *n* = 23 (38.3%) respondents, followed by *n* = 14 (23.3%) selecting 61–80% and *n* = 10 (16.7%) selecting 21–40% (Figure 1). When asked what proportion of their patients report following a ‘MS diet’, 0–20% was most frequently selected option by *n* = 32 (53.3%) respondents, followed by *n* = 17 (28.3%) selecting 21–40%, *n* = 6 (10.0%) selecting 41–60%. When asked what proportion of their patients with MS refuse disease-modifying therapies to pursue management of their MS with diet and lifestyle, 0–20% was most frequently selected option by *n* = 47 (78.3%) respondents, followed by *n* = 11 (18.3%) selecting 21–40%, and *n* = 1 selecting 41–60% and 61–80% each. When asked about the proportion of their patients with MS who have a cardiometabolic comorbidity, 21–40% was most frequently selected option by *n* = 18 (30.0%) respondents, followed by *n* = 16 (26.7%), selecting 41–60% and 61–80% each. When asked what proportion of their patients with MS have a nutrition-related issue, 0–20% was most frequently selected option by *n* = 32 (53.3%) respondents, followed by *n* = 14 (23.3%) selecting 21–40%, and *n* = 8 (13.3%) selecting 41–60%.

After excluding RDNs (*n* = 2), *n* = 45 (77.6%), respondents indicated that they have referred a patient with MS to an RDN (Table 1). Neurologists (92.6%) were more likely to report referring their patients with MS to RDNs compared to other non-RDN providers (64.5%; *χ*^2^ = 23.4, *p* = 0.02). Respondents who reported having referred to an RDN (*n* = 45) most frequently (*n* = 22, 48.9%) selected that RDNs are helpful for the care of patients with MS, followed by very helpful (*n* = 16, 35.6%; Table 1). Lack of nutrition knowledge/general healthy eating advice was selected as the most common reason for RDN referral by *n* = 34 (73.9%) respondents, which was followed by overweight/obesity (*n* = 31, 67.4%), and malnutrition (*n* = 13, 28.3%; Table 2). Neurologists were more likely to refer to RDNs compared to other providers for overweight/obesity (84.0% and 47.6%, respectively; *χ*^2^ = 6.87, *p* = 0.009). There were no differences between neurologists and other providers for any other reasons for referral.

Amongst respondents reporting reasons for not referring to an RDN (*n* = 13), the most common reasons reported were not having referral privileges at their institution (*n* = 5, 38.5%; all of which were not neurologists) and inadequate consultation time to refer to an RDN (*n* = 5, 38.5%), followed by being unsure how to refer to an RDN (*n* = 4, 30.8%; Table 2).

**Figure 2:**
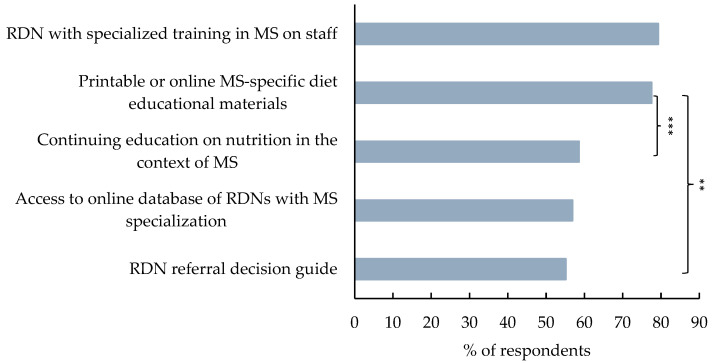
Reported resources that would be helpful to MS care providers and their patients. Bars represent the percent of participants selecting ‘yes’ for the corresponding option. Statistical significance is determined by chi-square test of independence and indicated by ** for *p* ≤ 0.01 and *** for *p* ≤ 0.001.

When the non-RDN respondents were asked if they or their patients with MS would benefit from additional resources, *n* = 46 (79.3%) selected having an RDN with specialized training in MS on staff at their institution, *n* = 45 (77.6%) selected having printable or online educational materials on diet that are specific to MS, and all other reasons were selected by over 50% (Figure 2). Respondents were more likely to select having printable or online educational materials specific to MS compared to having an RDN referral decision guide (*χ*^2^ = 10.7, *p* = 0.002) or having a continuing education on general nutrition in the context of MS for non-RDN healthcare providers (*χ*^2^ = 12.9, *p* < 0.001). There were no other differences observed between responses to additional resources.

## 4. Discussion

This pilot survey investigated the perceptions and utilization of RDNs in MS care among 60 multidisciplinary MS care providers and observed that more than 75% reported referring patients with MS to RDNs. MS care provider respondents most frequently attributed referrals to RDNs to lack of nutrition knowledge/general healthy eating advice and overweight/obesity among their patients with MS. The perception of RDNs as ‘helpful’ or ‘extremely helpful’ to the care of people with MS among most respondents emerged as a key finding and supports the high proportion of respondents who indicated that having an RDN with specialized training in MS on staff would be beneficial to their patients.

Neurologists were more likely to report referring to RDNs compared to other respondents, an observation which is likely driven by the lack of referral privileges reported by five non-neurologist respondents. According to Soelberg Sorensen et al., referring patients with need for dietetic services is considered the minimum requirements for multidisciplinary MS care units [34]. Obtaining referrals is a major barrier to healthcare service access experienced by people with MS [35]. Importantly, over 90% of neurologist respondents reported having ever referred a patient with MS to an RDN in the present study. Furthermore, nearly 80% of respondents reported that having an RDN with specialized training in MS on staff would benefit their patients, which would better represent the fully developed integrated multidisciplinary MS care unit design described by Soelberg Sorensen et al. [34]. Referring patients with MS would also help neurologists to circumvent their reported inadequate time for providing dietary advice to their patients with MS [7].

Results from this pilot survey suggest that diet remains of high interest to people with MS, as evidenced by the number of MS care provider respondents who indicated that a high proportion of their patients with MS inquire about diet. Respondents frequently selected having printable or online educational materials on diet that are specific to MS as a beneficial resource for their patients. Receiving educational handouts on diet from an MS care provider would meet the desire for diet resources and reduce confusion about where to seek dietary advice reported by people with MS [10,11,12,13]. However, this strategy would not provide the desired behavioral change support for facilitating dietary change and is generally less effective at improving diet quality compared to nutrition therapy provided by RDNs among people with MS [25,36,37]. Furthermore, since people with MS frequently use the internet for health information [8,10], this strategy may leave people with MS susceptible to extreme ‘MS diets’ [38] that are promoted online and often lack evidence [9]. This survey observed that 46.7% of respondents indicated that more than 20% of their patients follow a ‘MS diet’. These diets tend to be restrictive and may increase the risk of micronutrient deficiency, disordered eating, and malnutrition. Concerningly, neurologists report rarely referring patients with MS who follow restrictive diets to RDNs [7].

In addition, 21.7% of respondents in the present study indicated that more than 20% of their patients with MS decline disease-modifying therapies due to the perception that they can manage their disease with diet. While some diets may improve fatigue and quality of life [17], there currently is no evidence supporting that diet and lifestyle modifications can replace disease-modifying therapies for reducing MS disease activity and progression [14,15]; therefore, the inclusion of RDNs in MS care may help people with MS navigate the pitfalls of the dietary advice targeted to them [39]. A recent 2024 web-scrapping study observed that approximately 50% of online sources related to diet and MS recommend people with MS to consult with a RDN when making dietary changes [40] which is an improvement from the observations of online sources conducted in 2017 [9].

Over 80% of respondents who reported previously referring a patient with MS to an RDN indicated that RDNs are helpful or extremely helpful to the care of people with MS. This finding is supported by a survey of multidisciplinary MS care providers in Canada that observed over 60% of respondents reporting that RDNs are important or very important to MS care [30]. The effect of nutrition therapy by RDNs on the MS disease course remains sparse; however, preliminary trials have shown that it increases diet quality, improves micronutrient intake, and improves anthropometric measures among people with MS [23,24,25]. Whether these outcomes directly affect the course of MS on their own is unclear, but by reducing risk of cardiometabolic comorbidities, which are known risk factors for accelerated disease progression [41,42], nutrition therapy provided by RDNs may indirectly improve the course of MS [43].

Furthermore, beyond cardiometabolic comorbidities, there are several other nutrition-related conditions that are common among people with MS and that could benefit from nutrition therapy provided by an RDN. Neurogenic bowel dysfunction, including incontinence and constipation, affects between 39% and 73% of people with MS [44]. The first step in conservative therapy for neurogenic constipation is the optimization of fluid and fiber intake [45], which are interventions within an RDN’s scope of practice. Alarmingly, a 2023 meta-analysis found that the prevalence of dysphagia among people with MS is 44.8% [46]. The modification of diet texture and monitoring of nutritional status are essential elements of care for people with MS who have dysphagia [47]. The prevalence of malnutrition among people with MS is 15.5% according to the Global Leaders in Malnutrition diagnostic criteria [48], with dysphagia being one of the major risk factors among people with advanced MS [49]. It is concerning that, in the present study, 53.3% of respondents indicated that they thought 20% or fewer of their patients with MS had a nutrition-related issue and that malnutrition, bowel problems, and dysphagia were only reported as reasons for an RDN referral by 19.1 to 27.7% of respondents. Educating MS care providers to identify nutrition-related conditions may be an area to improve comprehensive MS care.

This study has several limitations. Firstly, since the response rate from the CMSC email listserv was extremely low, additional responses were acquired from other sources, which reduces the generalizability of our results to the broad community of MS care providers in the United States and Canada. Secondly, the collection of additional respondents via QR codes on posters and presentation slides may have increased selection bias in the sample by increasing the proportion of respondents with an interest in this topic. Third, due to the likely overlap of members of CMSC with participants in the CMSC and ACTRIMS Forum 2024 annual meetings, bias due to duplicate responses to this survey cannot be ruled out. Finally, this survey was designed to be brief and not burdensome to respondents, and as such many questions lack detail and important nuance. For example, nearly 80% of respondents indicated that they had referred a patient with MS to an RDN; however, information on the proportion of patients who received a referral, or the proportion of accepted referrals were not collected. The lifetime nature of this question also likely explains why the proportion of MS care providers who reported referring to an RDN is higher than reported in previous surveys [26,27,28,29,30].

## 5. Conclusions

This pilot survey of multidisciplinary MS care providers provides new insights into the perception and utilization of RDNs in MS care and justifies further exploration into the role of diet and RDNs in MS care from a larger sample of MS healthcare providers to gain a more comprehensive overview. Including RDNs on the MS care team is not only desired by MS care providers but could also benefit their patients with MS to make informed decisions regarding dietary modification.

## Figures and Tables

**Table 1:** Survey responses of multidisciplinary multiple sclerosis care providers ^1^.

Characteristic	Frequency (%) of Responses
Discipline (*n* = 60)	
Neurologist	27 (45.0)
Nurse	8 (13.3)
Nurse practitioner	6 (10.0)
Physical therapist	5 (8.3)
Occupational therapist	3 (5.0)
Pharmacist	2 (3.3)
Registered dietitian nutritionist	2 (3.3)
Other physician	2 (3.3)
Other providers ^2^	5 (8.3)
Have you ever referred a patient with MS to an RDN? (*n* = 58) ^3^	
Yes	45 (77.6)
No	10 (17.2)
Unsure/Cannot recall	3 (5.2)
How beneficial do you find RDNs to be to the care of people with MS? (*n* = 45) ^4^	
Extremely helpful	16 (35.6)
Helpful	22 (48.9)
Neither helpful nor unhelpful	5 (11.1)
Unhelpful	2 (4.4)
Extremely unhelpful	0

^1^ Abbreviations: multiple sclerosis, MS; registered dietitian nutritionist, RDN. ^2^ Includes one each of a psychologist, speech language pathologist, social worker, physiotherapist, and certified health education specialist. ^3^ Excludes RDNs. ^4^ Includes only those who reported referring to an RDN.

**Table 2:** Reasons for and for not referring to a registered dietitian nutritionist (RDN) reported by multidisciplinary MS care providers.

Characteristic	Frequency (%) of Responses
Reasons for RDN referral (*n* = 47) ^1^	
Lack of nutrition knowledge/general health eating advice	34 (72.3)
Overweight/obesity	31 (66.0)
Malnutrition	13 (27.7)
Bowel problems	11 (23.4)
Hyperglycemia	11 (23.4)
Dysphagia	9 (19.1)
Hypertension	8 (17.0)
Food allergy/sensitivity/intolerance	8 (17.0)
Unintentional weight loss	7 (14.9)
Hyperlipidemia	6 (12.8)
Lack of appetite	5 (10.6)
Micronutrient deficiency	3 (6.4)
Inflammation	1 (2.1)
Micronutrient toxicity	0
Reasons for not referring to a RDN (*n* = 13) ^2^	
Not having referral privileges	5 (38.5)
Inadequate consultation time	5 (38.5)
Unsure how to refer to RDN	4 (30.8)
Provide patients with handouts on healthy eating	3 (23.1)
RDN referrals should come from general practice physicians	2 (15.4)
Lack of evidence for role of diet in MS care	2 (15.4)
Lack of RDNs in area	1 (7.7)
Works in the in-patient setting	1 (7.7)

^1^ Includes only participants who reported referring to RDNs. ^2^ Includes only participants who reported not referring to RDNs.

## Data Availability

The data presented in this study are available on request from the corresponding author.

## Supplementary Materials

The following supporting information can be downloaded at: https://www.mdpi.com/article/10.3390/nu17030385/s1, Supplemental S1: Survey Questionnaire.
