# Supplementary material for: Perceptions and Utilization of Registered Dietitian Nutritionists in Multiple Sclerosis Care: A Pilot Survey of Multidisciplinary Providers
Source: Nutrients. 2025 Jan 22;17(3):385. doi: 10.3390/nu17030385 (PMC11820319; doi:10.3390/nu17030385)
Supplement: Supplementary file 1 [file nutrients-17-00385-s001.zip › nutrients-3398831-supplementary.pdf]

Supplemental S1: Survey questionnaire

1. **What is your role on the MS multidisciplinary care team?**
    1. Neurologist
    2. Nurse
    3. Nurse Practitioner
    4. Physician Assistant
    5. Occupational Therapist
    6. Physical Therapist
    7. Psychiatrist
    8. Neuro-ophthalmologist
    9. Medical assistant
    10. Pharmacist
    11. Psychologist
    12. Speech Language Pathologist
    13. Podiatrist
    14. Urologist
    15. Social Worker
    16. Registered Dietitian Nutritionist
    17. Physiotherapist
    18. Other Physician
    19. Other (please specify)
    20. I am not a healthcare provider

2. **Approximately what percentage of the people with MS you see in practice inquire about diet/nutrition advice?**
    1. 0-20%
    2. 21-40%
    3. 41-60%
    4. 61-80%
    5. 81-100%

3. **Approximately what percentage of the people with MS you see in practice follow a specific MS diet (Best Bet, OMS, Swank, Wahls, etc.)?**
    1. 0-20%
    2. 21-40%
    3. 41-60%
    4. 61-80%
    5. 81-100%

4. **Approximately what percentage of the people with MS you see in practice refuse DMTs due to the perception that they can manage their disease with diet/lifestyle/wellness/etc.?**
    1. 0-20%
    2. 21-40%
    3. 41-60%
    4. 61-80%
    5. 81-100%

5. **Approximately what percentage of the people with MS you see in practice have a cardiometabolic comorbidity (hypertension, hyperlipidemia, hyperglycemia, large waist circumference, heart disease)?**
    1. 0-20%
    2. 21-40%
    3. 41-60%
    4. 61-80%
    5. 81-100%

6. **Approximately what percentage of the people with MS you see in practice have a nutrition-related issue (malnutrition, micronutrient deficiency, food insecurity, lack of appetite, unintentional weight loss, slow/rapid gut motility, dysphagia, etc.)?**
    1. 0-20%
    2. 21-40%
    3. 41-60%
    4. 61-80%
    5. 81-100%

7. **Have you ever referred a person with MS to a Registered Dietitian Nutritionists (RDN)?** *Only show if Q1 ≠ 16*
    1. Yes
    2. No
    3. Unsure/can't recall

8. **Which reasons have you referred a patient with MS to a RDN? (If you are an RDN, please select the reasons you have received referrals) (Select all that apply).** *Only show if Q7 = 1 or Q1 = 16*
    1. Overweight/obesity
    2. Hypertension
    3. Hyperlipidemia
    4. Hyperglycemia
    5. Malnutrition

6. Micronutrient deficiency
7. Micronutrient toxicity
8. Lack of appetite
9. Unintentional weight loss
10. Dysphagia
11. Food allergy/sensitivity/intolerance
12. Lack of nutrition knowledge/General healthy eating advice
13. Bowel problems (constipation/bloating/diarrhea)
14. Other (Please specify)

9. How beneficial do you find RDNs to be to the care of people with MS? *Only show if Q3 = 1*

1. Extremely helpful
2. Helpful
3. Neither helpful nor unhelpful
4. Unhelpful
5. Extremely unhelpful

10. What are your top reasons for not referring people with MS to a RDN? (Select up to three) *Only show if Q3 ≠ 1*

1. Unsure how to refer to a RDN
2. Inadequate consultation time to discuss
3. Lack of evidence for the role of diet in MS management
4. RDNs do not receive adequate training
5. Referral to RDNs should come from a patients' general physician
6. I do not have referral privileges at my practice
7. I provide my patients with educational handouts on a healthy diet
8. Other (Please explain)

11. Would you, your patients, and/or your practice benefit from the following?

1. A continuing education on the general nutrition for non-RDNs
2. Printable/online resources on a healthy diet to provide to patients
3. Having an RDN with specialized training in MS care on staff at your practice
4. An online database for finding local/remote RDNs for patient referrals
5. A 'best practices' document for RDN referral decision making
6. Other (Please explain)
